# Alternative Pathways of Acetogenic Ethanol and Methanol Degradation in the Thermophilic Anaerobe *Thermacetogenium phaeum*

**DOI:** 10.3389/fmicb.2019.00423

**Published:** 2019-03-19

**Authors:** Anja Keller, Bernhard Schink, Nicolai Müller

**Affiliations:** ^1^Department of Biology, Universität Konstanz, Konstanz, Germany; ^2^Konstanz Research School Chemical Biology, Konstanz, Germany

**Keywords:** *Thermacetogenium phaeum*, methanol, ethanol, ethanolamine, acetate, syntrophy, anaerobic metabolism, acetogenesis

## Abstract

Growth of the anaerobic thermophile *Thermacetogenium phaeum* with methanol, ethanol, ethanolamine, and acetate was investigated in axenic cultures and in syntrophic cultures with *Methanothermobacter thermautotrophicus*. Microcompartment genes were identified in the *T. phaeum* genome, and presence of microcompartments was confirmed by transmission electron microscopy and proteome analysis. These genes were expressed only during growth with ethanolamine. Proteome data were compared after growth with all four substrates, and activities of key enzymes of the Wood–Ljungdahl pathway and of enzyme systems leading to production or degradation of acetaldehyde such as alcohol dehydrogenase, aldehyde:ferredoxin oxidoreductase, acetate kinase, and phosphate acetyltransferase were measured in cytoplasmic fractions. Accounting of fermentation stoichiometries and growth yields with all four substrates showed that ethanol and methanol oxidation follow the same stoichiometries as in *Acetobacterium woodii*. On the other hand, the pathways of ethanol and methanol degradations vary between both organisms. Growth yields of *T. phaeum* were substantially lower than reported for *A. woodii*. Since *T. phaeum* has no Rnf complex encoded in its genome, the mechanisms of ATP synthesis have to be different from those of *A. woodii*. In addition to the central degradation pathways also found in *A. woodii, T. phaeum* maintains enzyme systems that compensate for the absence of an Rnf-complex but which on the other hand cause a loss of energy. On the basis of our data, pathways of methanol and ethanol degradation in *T. phaeum* are discussed.

## Introduction

*Thermacetogenium phaeum* is a strictly anaerobic, thermophilic bacterium that grows with a broad spectrum of organic substrates including primary alcohols and methylated compounds (Hattori et al., 2000). Like its next relative, the mesophilic bacterium *Syntrophaceticus schinkii* (Westerholm et al., 2010), it is able to oxidize acetate in syntrophic co-culture with *Methanothermobacter thermautotrophicus*. Besides these two strains, only the mesophilic bacterium *Clostridium ultunense* (Schnürer et al., 1996), the thermophilic bacterium *Pseudothermotoga lettingae* (Balk et al., 2002), the strain AOR (Lee and Zinder, 1988), *Tepidanaerobacter acetatoxydans* (Westerholm et al., 2011) and ‘*Candidatus Syntrophonatronum acetioxidans*’ (Timmers et al., 2018) were described to oxidize acetate syntrophically. The most thoroughly investigated pathway of anaerobic acetate oxidation is the reversed Wood–Ljungdahl pathway. The key enzyme of this pathway is the bifunctional CO-dehydrogenase/ acetyl-coenzyme A (CoA) synthase. The reaction catalyzed by this enzyme is reversible; it is able to form C-C bonds and to cleave them (Ragsdale, 2007; Ragsdale and Pierce, 2008). The Wood–Ljungdahl pathway is used not only during acetate oxidation and acetogenic growth with H_2_/CO_2_ but also in the conversion of methyl groups to acetate (Drake and Daniel, 2004).

The biochemistry of acetogenic growth with H_2_/CO_2_, methanol (Bache and Pfennig, 1981; Kremp et al., 2018), ethanol (Bertsch et al., 2016), 1,2-propandiol (Schuchmann et al., 2015), and ethylene glycol (Trifunović et al., 2016) has been elucidated in detail with *Acetobacterium woodii*. In this bacterium, a sodium ion-dependent Rnf complex plays a central role to reduce ferredoxin with electrons derived from NADH oxidation (Biegel et al., 2009, 2011; Biegel and Müller, 2010). Such an Rnf complex is not found in the genome of *T. phaeum* (Oehler et al., 2012), thus, the energy metabolism of this bacterium must be essentially different from that of *A. woodii*.

Another well-studied acetogenic model organism is *Moorella thermoacetica*. Here, the mechanism of energy conservation has not yet been elucidated completely. Additionally, *M. thermoacetica* has no Rnf complex encoded in its genome, but contains cytochromes and menaquinone (Gottwald et al., 1975), which could play a role in electron transport. A bifurcating hydrogenase using simultaneously NAD^+^ and oxidized ferredoxin as electron acceptors was purified and appears to be the only reaction system yielding reduced ferredoxin during growth with H_2_/CO_2_ as sole substrate (Wang et al., 2013). An Ech hydrogenase was described to be expressed, but its function in energy conservation has not yet been clarified (Wang et al., 2013). *M. thermoacetica* also grows on methanol (Daniel et al., 1990) and ethanol, but here the pathways have not yet been investigated (Drake and Daniel, 2004; Jiang et al., 2009).

The energy metabolism of *T. phaeum* has not yet been studied in as much detail as that of *A. woodii* and *M. thermoacetica*. In 2012, the genome of *T. phaeum* was sequenced (Oehler et al., 2012). As the reverse Wood–Ljungdahl pathway of acetate oxidation does not yield net ATP by substrate-level phosphorylation (one ATP is needed for the phosphorylation of acetate to acetyl-phosphate and one ATP is produced during conversion of formyl-THF to formate), a membrane-bound electron transport system must be responsible for energy conservation (Schuchmann and Müller, 2014). As a membrane-bound Rnf complex is missing in *T. phaeum*, the only known potentially proton-translocating enzyme complex encoded in the genome is Ech hydrogenase. However, a convincing concept of energy conservation is missing so far, and the current study describes several genes that are induced during syntrophic acetate oxidation that are potentially involved in energy conservation.

The biochemical pathways underlying the degradation of ethanol, methanol, and ethanolamine in *T. phaeum* have not been investigated either and are the focus of this study. Ethanolamine degradation is known from other organisms such as *Enterococcus faecalis* or *Salmonella typhimurium* and occurs in microcompartments (Kerfeld et al., 2018). Microcompartments are primitive organelles in bacteria that are composed of proteinaceous shell proteins that contain metabolic enzymes, in the case of ethanolamine degradation alcohol dehydrogenase, acetaldehyde dehydrogenase, and ethanolamine ammonia lyase. It is assumed that microcompartments protect the cell from toxic intermediates such as aldehydes or provide a concentration mechanism for a certain metabolite, i.e., CO_2_ for RubisCo (Kerfeld et al., 2018). In the present study, total proteome analyses were performed with *T. phaeum* grown with methanol, ethanol, ethanolamine, or acetate to inventory all potential enzyme systems involved in its energy metabolism. The presence of key enzymes, particularly of aldehyde:ferredoxin oxidoreductase, alcohol dehydrogenase, acetate kinase, phosphate acetyltransferase, CO-dehydrogenase, and hydrogenase, were investigated by activity assays. As part of the proteome analysis, the abundance of microcompartments was also investigated under the different growth conditions.

## Materials and Methods

### Origin of Organisms and Cultivation Conditions

The axenic culture of *T. phaeum* strain PB (DSMZ 26808) and the co-culture with *Methanothermobacter thermautotrophicus* strain TM (DSM 12269) as methanogenic partner organism as well as *A. woodii DSM 1030 were o*btained from the German Culture Collection DSMZ (Braunschweig, Germany). Cultures of *T. phaeum* and *M. thermoautotrophicus* were grown at 55°C in the dark in freshwater mineral medium modified after DSMZ medium No. 880 (Hattori et al., 2000). Trace element solution SL10 was used (Widdel et al., 1983), and 0.15 g/l Na_2_S was used as reducing agent instead of 0.3 g/l. The pH was adjusted to 6.8 for growth with acetate, acetaldehyde (AcAld) and ethanolamine (EA), and to 7.2 for growth with methanol (MeOH) and ethanol (EtOH). Substrates were added from filter-sterilized stock solutions directly before inoculation; the acetate stock solution was autoclaved. Cultures were inoculated into growth medium at a ratio of 1:10 (v/v). Cultures used in this study were transferred to fresh media with the respective substrate at least 10 times for complete adaptation to the respective growth condition. For growth experiments, all substrates were added to 40 mM final concentration; acetaldehyde was added to 5 mM final concentration. Growth experiments were conducted with seven replicates in 25 ml tubes filled with 9 ml medium and 1 ml inoculum. Four tubes were used for the growth curves monitoring the time courses of OD_600_, and for sampling of gaseous and dissolved metabolites. The remaining three samples were used only to determine accurate start and end values for calculating electron balances. Growth experiments with acetaldehyde were performed in 150 ml bottles filled with 45 ml medium and 5 ml inoculum as growth was weak and unreliable in 25 ml test tubes. *A. woodii DSM 1030 was* cultivated in bicarbonate-buffered, sulfide-reduced anoxic medium as described elsewhere (Patil et al., 2015). Fructose was added at a concentration of 10 mM as a source of carbon and energy, and 0.2% (w/v) yeast extract was added as growth-stimulating supplement. Cultures were incubated at 30°C in the dark.

### Analytical Methods to Monitor Growth and Substrate Turnover

Optical densities were monitored at 600 nm with a Camspec M107 spectrophotometer (Leeds, United Kingdom) fitted with a tube holder to measure optical densities directly in culture tubes. The optical density of cultures growing with acetaldehyde was measured in plastic cuvettes at the same wavelength with a Jenway 6300 spectrophotometer (Staffordshire, United Kingdom).

Substrate depletion and product formation were monitored by HPLC using an isocratic Shimadzu system with a LC-10AT vp pump and an RID detector (RID-10A, Shimadzu, Tokyo, Japan). Flow rate was 0.6 ml per min with 5 mM H_2_SO_4_ as eluent. Compounds were separated on a Rezex^TM^ RHM-Monosaccharide H^+^ (8%) ion exchange resin column (LC column 300 mm × 7.8 mm, 00H-0132-K0, Phenomenex, Los Angeles, CA, United States) at 60°C. To detect ethanolamine depletion, a derivatization reaction was used as described (Sanger, 1945; Denger et al., 1997). The reaction mixture containing 0.066% (w/v) 1-Fluoro-2,4-dinitrobenzene, 82 mM NaHCO_3_ and 10 μl sample was incubated for 2 h at 30°C at a shaking speed of 300 rpm. To avoid complete saturation of the reagents, the start sample (taken directly after inoculation and substrate addition) was diluted 1:2 with carbonate buffer. The reaction was stopped by addition of 50 μl 37% HCl. The derivative was detected with a UV detector (SPD-M20A, Shimadzu, Tokyo, Japan) at 360 nm. Separation of the sample by HPLC was achieved with a C18 column [Eurospher 125 mm × 3 mm (12CE181ESJ), Knaur, Berlin, Germany]. The analysis run started with 3 min of 20% acetonitrile in 1% formic acid, followed by a linear gradient over 22 min to a final concentration of 80% acetonitrile, and a subsequent washing step with 20% acetonitrile for 10 min.

Methane and H_2_ were measured in 500 μl samples of the culture headspace by GC (SGI 8610C, SRI Instruments, Los Angeles, CA, United States). Gases were separated on a 3 m Hayesep-D column (60°C, carrier gas N_2_). CH_4_ was detected with a flame ionization detector (135°C) and H_2_ with a thermal conductivity detector (150°C). The detection limits for H_2_ and CH_4_ were 0.02 mM (0.05%) and 0.04 mM (0.1%), respectively. The software PeakSimple v4.44 was used to record the chromatograms.

### Preparation of Cell-Free Extract and Subcellular Fractions

Co-cultures were harvested by centrifugation at 7,010 × *g* for 15 min at 4°C. The pellet was washed with 50 mM Tris-HCl buffer, pH 7.5, containing 3 mM dithiothreitol (DTT), and suspended in 1 ml of the same buffer per l culture. The suspended pellet was then separated by density gradient centrifugation, modified after (Luo et al., 2002; Enoki et al., 2011). A self-assembly gradient with 70% Percoll in double-distilled water containing sucrose at a final concentration of 250 mM was centrifuged in a Type 70-Ti rotor in an Optima LE-80K Ultracentrifuge (Beckman Coulter, Brea, CA, United States) at 45,000 × *g*. The centrifugation step was shortened to 1 h to obtain a sharp band of *T. phaeum* cells. The upper band containing *T. phaeum* cells was washed with 50 mM Tris-HCl, pH 7.5, containing 3 mM DTT. The pellet was suspended in 2 ml of the same buffer and either used directly to prepare cell-free extracts for enzyme assays or frozen in liquid nitrogen and stored at -20°C to be used for proteome analysis. Axenic cultures of *T. phaeum* or *A. woodii* were harvested according to the same protocol but without density gradient centrifugation.

Cells were disrupted by at least three passages through a French Pressure Cell (Aminco, Silver Spring, MD, United States) which was pre-chilled on ice and operated at 137 MPa pressure. The suspension was centrifuged at 11,337 × *g* for 5 min to remove cell debris and unopened cells. Subcellular fractionation was done by ultracentrifugation for 1 h at 100,000 × *g* in an Optima TL-ultracentrifuge using a TLA110-rotor (Beckman Coulter, Brea, CA, United States). The supernatant was defined as cytoplasmic fraction and the pellet was washed once in 50 mM Tris-HCl, pH 7.5, containing 3 mM DTT. After an additional centrifugation step, the pellet was suspended in 0.5 ml buffer and defined as the membrane fraction. Cell-free extracts to be used for enzyme activity measurements were prepared under strictly anoxic conditions in an anoxic glove box (Coy, Ann Arbor, MI, United States). Buffers were made anoxic by alternatively applying vacuum and 100% N_2_ at least three times to serum bottles sealed with rubber stoppers.

### Mass Spectrometry

Cell-free extracts from axenic cultures grown with methanol, ethanol, or ethanolamine and syntrophic cultures grown with ethanol, ethanolamine, or acetate were separated into membrane and cytoplasmic fractions as described before. To remove interfering lipids, the membrane fractions were suspended in 10% SDS, let run 2 cm into a 12% SDS gel (Laemmli, 1970) stained with colloidal Coomassie (Neuhoff et al., 1988; Schmidt et al., 2013), and excised. After trypsin digestion, samples were analyzed by liquid chromatography nanospray tandem mass spectrometry (LC-MS/MS). An LTQ-Orbitrap mass spectrometer (Thermo Fisher) and an Eksigent nano-HPLC were used with a reversed-phase LC column (5 μm, 100 Å pore size C18 resin in a 75 μm i.d. × 15 cm long piece of fused silica capillary, Acclaim PepMap100, Thermo Scientific). After sample injection, a washing step with 10% mobile phase B (0.1% formic acid in acetonitrile) and 90% mobile phase A (0.1% formic acid) was applied for 5 min at a flow rate of 300 nl/min. Subsequently, a linear gradient of 10% mobile phase B to 35% mobile phase B in 95 min was applied to elute the peptides. A washing step of 5 min (35 to 80% B) followed. To operate the LTQ-Orbitrap mass spectrometer, the data-dependent mode was used. Ten MS/MS scans were performed after each full MS scan (30,000 resolving power). After dynamically selecting the 10 most abundant molecular ions, they were fragmented by collision-induced dissociation (CID). Normalized collision energy of 35% was used in the LTQ ion trap, and a dynamic exclusion was allowed. A protein database of *T. phaeum* was searched against tandem mass spectra using Mascot (Matrix Science) with “Trypsin” enzyme cleavage, static cysteine alkylation by chloroacetamide and variable methionine oxidation. For semi-quantitative analysis of relative protein abundance, the area values of the respective peaks in the ion chromatogram analyzed by the Proteome Discoverer software (Thermo Fisher) were used.

### Enzyme Activity Measurements

If not stated otherwise, enzyme activities were assayed in the cytoplasmic fractions at least in triplicates with a Jasco V630 or V730 spectrophotometer (Tokyo, Japan). All enzyme assays were conducted in anoxic 50 mM Tris-HCl buffer, pH 7.5, with 3 mM DTT at 55°C. The continuously measured photometric assays were performed in cuvettes sealed with rubber stoppers and previously flushed with 100% nitrogen.

CO dehydrogenase activity was assayed according to Diekert and Thauer (1978) with 1 mM benzyl viologen (BV) or methyl viologen (MV) as electron acceptor. The reaction was started by injection of 100 μl CO (100%) into the headspace. Hydrogenase was measured in an analogous manner; here the reaction was started with injection of 100 μl H_2_ (100%). The reduction of artificial electron acceptors was monitored photometrically [BV: 𝜀_578_ = 8.65 mM^-1^ cm^-1^ (McKellar and Sprott, 1979), MV 𝜀_578_ = 9.7 mM^-1^ cm^-1^ (Daniels et al., 1977; Diekert and Thauer, 1978)]. Formate dehydrogenase was measured with 1 mM benzyl viologen, 1 mM methyl viologen or 0.25 mM NAD^+^ as electron acceptor [modified after (Schmidt et al., 2014)]. The reaction was started by adding 5 mM sodium formate. NADH formation was monitored at 340 nm [𝜀 = 6.292 mM^-1^⋅cm^-1^ (Ziegenhorn et al., 1976)], benzyl viologen or methyl viologen reduction both at 578 nm. Alcohol dehydrogenase was assayed in the oxidative direction according to Schmidt et al. (2014) but the reaction was started with 50 mM ethanol (Schmidt et al., 2014). Non-acetylating acetaldehyde:acceptor oxidoreductase was measured in the oxidative direction with either 1 mM benzyl viologen, 1 mM methyl viologen or 0.25 mM NAD^+^ [modified after (Hensgens et al., 1995)]. The reaction was started with 0.5 mM acetaldehyde. Acetylating acetaldehyde dehydrogenase was measured analogously to the acetaldehyde:acceptor oxidoreductase with 0.25 mM NAD^+^ but the reaction was started by addition of 0.33 mM coenzyme A. Phosphate acetyltransferase was measured according to Bergmeyer et al. (1963); Bergmeyer (1974), Müller et al. (2008) but the reaction was started with 3 mM acetyl phosphate. Acetate kinase was measured with modifications according to Nishimura and Griffith (1981) and Müller et al. (2008). The assay was performed at 55°C under oxic conditions. Samples of 300 μl were taken from the reaction mix after 0, 5, and 10 min and mixed with 200 μl of a freshly prepared neutralized 2.5 M hydroxylamine (pH 5.4) solution. After incubation for 10 min at room temperature, 300 μl of a 1.6% w/v FeCl_3_ solution in 1 M HCl and 4% (w/v) trichloroacetic acid was added. The absorption was measured at 540 nm and compared to a calibration curve of various concentrations of acetyl-P prepared with the same solutions. Methylene-Tetrahydrofolate (THF) reductase was assayed with 1 mM benzyl viologen or 0.25 mM NADH and cytoplasmic fraction or membrane fraction. Additionally, the MTHFR activity was enriched via an anion exchange column (HiTrapQ HP column, 5 ml, GE Healthcare, Pittsburgh, PA, United States), operated manually with syringes and by stepwise eluting bound protein. All purification steps were carried out under strictly anoxic conditions in an anoxic chamber (Coy, Ann Arbor, MI, United States). After application of the cytoplasmic fraction, the column was washed with five column volumes of buffer (Tris-HCl, 50 mM, pH 7.5, 3 mM DTT). Fraction 1 was eluted with 10 ml of 50 mM Tris-HCl buffer, pH 7.5, 3 mM DTT with 200 mM NaCl and thereafter, fraction 2 was eluted with 10 mL of the same buffer with 1 M NaCl. The assay with benzyl viologen as electron acceptor was started by addition of 0.2 mM methyl-THF (modified after (Clark and Ljungdahl, 1984; Bertsch et al., 2015). The assay with NADH as electron donor was started by addition of NADH, and NADH oxidation was monitored at 365 nm [𝜀 = 3.441 mM^-1^⋅cm^-1^ (Ziegenhorn et al., 1976)]. Methylene-THF was synthesized freshly and directly in the buffer by addition of 1.5 mM formaldehyde and 0.5 mM THF (Bertsch et al., 2015). This mixture of buffer and methylene-THF was considered to be stable for 1 day. As this reaction mix contains formaldehyde, a control with only formaldehyde was performed. Methylene-THF dehydrogenase was measured with 0.25 mM NAD^+^ as electron acceptor in fractions 1 and 2 of the purified cytoplasmic fraction and NADH formation was monitored at 365 nm [𝜀 = 3.441 mM^-1^⋅cm^-1^ (Ziegenhorn et al., 1976)].

### Transmission Electron Microscopy (TEM)

For transmission electron microscopy (TEM), cells grown syntrophically on ethanol, ethanolamine or acetate and axenic cultures grown with methanol were harvested in the late log phase. Two 20 ml samples of each culture were centrifuged (2,500 × *g*) and the pellets were either fixed for 45 min with 2.5% glutardialdehyde in 0.1 M HEPES buffer, pH 7.0, or with additional 0.15% ruthenium red at 55°C under anoxic conditions. After pre-fixation, samples were centrifuged (4,000 × *g*) and the supernatants were removed. Pellets were embedded in 1.5% agarose in 0.05 M HEPES, pH 7.0. The pellets were submersed in fresh fixative for 5 h at 0°C, rinsed three times for 10 min each in 0.1 M HEPES buffer, pH 7.0, and treated with 2% OsO_4_ in 0.1 M HEPES, pH 7.0, for 1 h at 4°C followed by three more washing steps. For dehydration, samples were treated first with 30% (10 min) and then with 50% (15 min) pre-chilled ethanol (4°C). Samples were stained overnight at 4°C with a saturated solution of uranyl acetate in 70% ethanol. To this step, one of the two subsamples per culture condition was always treated with additional 0.05% ruthenium red added to the described buffers. The dehydration was completed by a sequence of washing steps at room temperature (3 min × 10 min in 70, 80, 90, 96, 100% ethanol). The last step was repeated with dried ethanol for 1 h before samples were infiltrated with Spurr’s resin by a graded series with increasing amounts of resin dissolved in pure ethanol [12.5% (1 h), 25% (2 h), 50% (2 h) 75% (overnight), pure Spurr (2 h × 2 h)]. The polymerization was performed for 48 h at 65°C. Ultrathin sections (50 nm thickness) were cut from the blocks using freshly prepared glass knives or diamond knives (diatome 45°, Biehl, Switzerland) in a Leica UC7 ultramicrotome (Leica, Wetzlar, Germany). Pictures were taken with a TRS Tröndle slow-scanning digital camera (TRS, Munich, Germany) connected to a Zeiss EM Leo 912 Omega (Zeiss, Oberkochen, Germany) at an accelerating voltage of 80 kV and 40,000-fold magnification.

## Results

### General Properties of *T. phaeum* During Growth on Different Substrates

*Thermacetogenium phaeum* grew exponentially with all substrates (Supplementary Figure 1). Exponential growth phases were used to calculate the growth rate constants and doubling times (Table 1). The lowest doubling time with 2.8 h was observed during syntrophic growth with ethanolamine. The highest doubling time with 42 h was observed during syntrophic growth with acetate. This slow growth was also reflected in the time needed to consume the substrate completely. Syntrophic cultures grown with acetate needed 21 days to reach stationary phase, while stationary phase was reached already after 6 days when grown with ethanolamine. Growth in axenic culture with ethanol or ethanolamine was poorer than in syntrophic co-culture. During growth with ethanol, the OD of the axenic culture increased to a maximum of 0.12 while the syntrophic culture reached a maximum OD of 0.28. The axenic culture stopped growing with ethanol at a final ethanol concentration of about 13 mM. The same was observed for growth with ethanolamine. Here the final ethanolamine concentration was 8 mM, the final ethanol concentration 6 mM and the increase in OD was 0.19 in axenic and 0.36 in syntrophic cultures. Syntrophic cultures converted ethanol completely to acetate and methane, and ethanolamine was depleted to a concentration of 5 mM. Syntrophic cultures with methanol exhibited no significant growth difference compared to axenic methanol-grown cells (data not shown). In these syntrophic methanol cultures, methane accumulated to a maximum of 1.5 mM in the culture headspace while maximally 23 mM acetate was formed, indicating that acetogenic methanol degradation was the dominating process and outcompeted methanogenesis. Growth with acetaldehyde was observed in axenic and syntrophic cultures of *T. phaeum*. However, growth depended on the presence of 3 mM L-cysteine in the medium and stopped after transfer to cysteine-free medium. Cultures with 3 mM cysteine as sole substrate exhibited no growth after one transfer in this medium (data not shown).

**Table 1:** Stoichiometry of degradation and growth yields of *T. phaeum* cultures in axenic and syntrophic cultures.

	Substrate degraded (mM)	Cell dry weight formed (mg/l)^a^	Substrate assimilated (mM)^b^	Products formed (mM)				Growth yield (g/mole substrate)	Electron recovery (%)^d^	Doubling time (h)
				Acetate	Ethanol	CH_4_	H_2_			
**Axenic culture**										
Ethanol	27 ± 3^c^	31 ± 5	0.4 ± 0	35 ± 2	bd	bd	<0.5	1.2 ± 0	89 ± 10	32.0
Ethanolamine	30 ± 1	46 ± 3	0.8 ± 0	32 ± 1	3 ± 2	bd	<0.5	1.6 ± 0	101 ± 9	15.0
Methanol	40 ± 0^c^	160 ± 3	4.4 ± 0	27 ± 3	bd	bd	bd	4.5 ± 0	100 ± 10	12.5
**Syntrophic culture**										
Ethanol	39 ± 0^c^	69 ± 2	1 ± 0	45 ± 4	bd	26 ± 0	bd	1.8 ± 0	125 ± 6	11.0
Ethanolamine	32 ± 0	91 ± 2	1.5 ± 0	35 ± 1	bd	16 ± 1	bd	3.0 ± 0	133 ± 1	2.8
Acetate	37 ± 3	93 ± 2	1.9 ± 0	bd	bd	28 ± 1	bd	2.6 ± 0	79 ± 9	42.4

*All growth experiments were carried out in triplicates, bd: below detection limit, 1 mM for acetate, 1 mM for ethanol, 0.04 mM for CH_4,_ 0.02 mM for H_2_, 1 mM ethanolamine, 1 mM methanol. ^*a*^Cell dry weight was calculated using the assumption of 250 mg/l dry weight for OD_600_ = 1. ^*b*^Assimilation of substrate was calculated with the theoretical formula < C_4_H_7_O_3_> for cell mass. Assimilation equations: Ethanol: 17 C_2_H_7_O + 14 H_2_CO_3_ → 12 < C_4_H_7_O_3_> + 23 H_2_O; Ethanolamine: 17 C_2_H_7_ON + 6 H_2_CO_3_ → 10 < C_4_H_7_O_3_> + 5 H_2_O + 17 NH_3_; Methanol: 17 CH_3_OH + 7 H_2_CO_3_ → 6 < C_4_H_7_O_3_> + 20 H_2_O. ^*c*^Assumed substrate concentration added equals 40 mM. ^*d*^Electron balances were calculated by summing up the total amounts of electrons on the product and substrate side and calculating the percentage of electrons released on the product side compared to the substrate electrons. For calculation of total electrons, formal complete oxidation of substrates and products to CO_2_ or protons for H_2_ was assumed and the respective molar amounts of electrons calculated: ethanol oxidation to CO_2_ yields 12 mol e^-^/mol ethanol, ethanolamine 10 e^-^, methanol 6 e^-^, acetate 8 e^-^, methane 8 e^-^, hydrogen 2 e^-^.*

### Total Proteome Analysis

#### Wood–Ljungdahl Pathway

Total proteome analysis revealed the presence of the key enzymes of the Wood–Ljungdahl pathway under all growth conditions (Supplementary Figure 2). The CODH/ACS precursors and their maturation factors (Tph_c15140–Tph_c15190), the methyltetrahydrofolate-corrinoid iron–sulfur protein Co-methyltransferase (Tph_c15130) and the methylene-tetrahydrofolate (THF) reductase (Tph_c15100–Tph_c15110) are encoded in one gene cluster, whereas the methylene-THF dehydrogenase (Tph_c16310–Tph_c16320) is encoded further upstream. All these genes are expressed during growth with all substrates. The two methylene-THF reductase subunits [Tph_c15100 (metF) and Tph_c15110 (metV), 313 and 223 amino acids respectively] are 39 and 34% identical to the methylene-THF reductase subunits in *A. woodii* (metF 298 amino acids and metV 205 amino acids). However, the neighboring third subunit annotated as electron transport complex protein RnfC2 in *A. woodii* is missing in *T. phaeum*. The genome contains two formate-THF ligases (Tph_c08280 and Tph_c26780) which were both expressed during growth with all substrates (Supplementary Figure 2).

#### Hydrogenases and Formate Dehydrogenases

All five hydrogenases encoded in the genome (Oehler et al., 2012) were found to be expressed. The Ech hydrogenase (Tph_c21310-21360), a periplasmatic [NiFeSe] hydrogenase (Tph_c06350- 06370) and a non-F_420_ reducing hydrogenase (Tph_c26910-26930) were constitutively expressed during growth with all substrates (Supplementary Figure 3). One hydrogenase is located in a formate hydrogenlyase system (Tph_c26250- 26370) and was highly induced during growth in axenic culture with ethanolamine and ethanol. Taken together, the areas of protein abundances of ethanolamine and ethanol grown cells made up more than 92% of the sums of area of all 10 individual proteins of this cluster measured by mass spectrometry (Supplementary Figure 3). In other words, the summed areas of protein abundances, for example for the formate hydrogenlyase subunit 5 (Tph_c26330), measured by mass spectrometry correspond to 100 and 48% of the area corresponds to cells grown with ethanol while 44% are attributed to cells grown with ethanolamine. The fifth hydrogenase is an NAD(P)-dependent iron-only hydrogenase (Tph_c18430- 18460). The genes in this cluster were slightly induced during growth with methanol, making up between 47 and 60% of the sums of area of all individual proteins of this cluster.

The formate dehydrogenase gene (Tph_c18420), which is located in the same gene cluster, was also slightly induced during growth with methanol. Two more formate dehydrogenase genes are located next to a putative NADH:quinone oxidoreductase (Tph_c21680- 21660, Tph_c08060- 08040). Another formate dehydrogenase gene (Tph_c27290) is located next to a molybdopterin-binding protein and a 4Fe–4S cluster-binding protein. It was expressed only during syntrophic growth with ethanol and ethanolamine. During syntrophic growth with acetate, a putative membrane-bound formate dehydrogenase was induced that contains four subunits (Tph_c15380-15410). A total of 29 to 74% of the sums of area of all individual proteins of the subunits measured by mass spectrometry originates from cells grown with acetate (Supplementary Figure 3). In the same cluster, a sec-independent protein translocase and two heterodisulfide reductases are located as well.

#### Alcohol Dehydrogenases and Acetaldehyde Oxidoreductases

**FIGURE 1:**
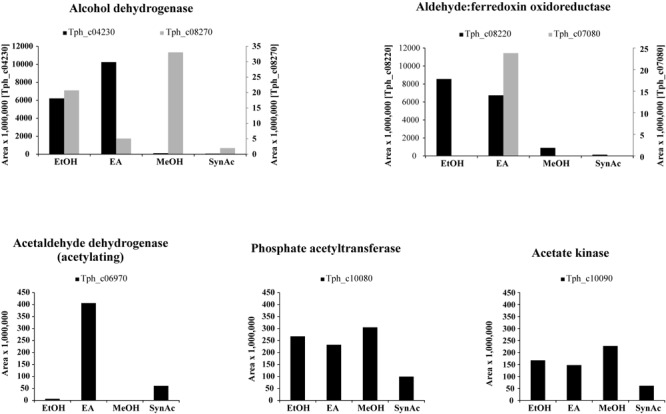
Bar plots depicting the proteome data of selected enzymes related to acetaldehyde metabolism. Y-axes give area values multiplied by 1,000,000 to obtain better visualization. EA, axenic growth with ethanolamine; EtOH, axenic growth with ethanol; MeOH, axenic growth with methanol; SynAc, syntrophic growth with acetate.

Total proteome analysis revealed that acetate kinase (Tph_c10090) and phosphate acetyltransferase (Tph_c10080) were expressed constitutively under all growth conditions (Figure 1). Four alcohol dehydrogenases (Tph_c04230, Tph_c04260, Tph_c08270, and Tph_c18280) and one acetylating acetaldehyde dehydrogenase (Tph_c06970) are encoded in the genome. The acetylating acetaldehyde dehydrogenase is located in a gene cluster with other enzymes responsible for ethanolamine degradation and was induced in cells grown with ethanolamine (Supplementary Figure 6). Three of the alcohol dehydrogenases are located next to NADH:quinone oxidoreductases (Tph_c04200- 04260, Tph_c08210- 08270) and were induced during growth with ethanolamine and ethanol (Supplementary Figure 4). The fourth alcohol dehydrogenase was not expressed during growth with any substrate. In total, six aldehyde:ferredoxin oxidoreductases are encoded in the genome (Tph_c04180, Tph_c07080, Tph_c08220, Tph_c19480, Tph_c20350, and Tph_c27630). Two of these aldehyde:ferredoxin oxidoreductases are completely identical homologs (Tph_c08220, Tph_c04180) and could not be differentiated by proteome analysis (Supplementary Figure 4). In this study, we decided to assign the proteomic data to the locus tag Tph_c08220. Proteins annotated as Fe-S-cluster-containing hydrogenase component 2 are located adjacent to these aldehyde:ferredoxin oxidoreductases. Additionally, these proteins are completely identical homologs. The third aldehyde:ferredoxin oxidoreductase (Tph_c07080) was expressed only during syntrophic and axenic growth with ethanolamine (Supplementary Figure 6). The latter three were not expressed under any growth condition.

#### Glycine Cleavage System

It was proposed that the glycine cleavage system plays a role in acetate oxidation in the thermophile *Syntrophaceticus schinkii*, the closest relative of *T. phaeum* (Westerholm et al., 2010). One of the enzymes involved, a serine ammonia lyase, is encoded in the genome of *T. phaeum* (Tph_c17140, Tph_c17150) but was not identified in the proteome after growth with acetate. Serine hydroxymethyltransferase (Tph_c27490) was expressed but not specifically induced during syntrophic growth with acetate (Supplementary Figure 5). Enzymes of the glycine cleavage system were partly expressed but never induced during growth with acetate. The dihydrolipoamide dehydrogenase (Tph_c17180) and glycine dehydrogenase beta subunit (Tph_c17190) were expressed during growth with acetate but the glycine dehydrogenase alpha subunit (Tph_c17200) was not. The aminomethyltransferase (Tph_c17220) and the cleavage protein H (Tph_c17210) were not expressed during growth with acetate.

#### Bacterial Microcompartments (BMCs)

The genome of *T. phaeum* contains an ethanolamine utilization (*eut*) cluster consisting of 20 genes, of which 18 were strongly induced during syntrophic or axenic growth with ethanolamine (Supplementary Figure 6). The *eut* cluster contains not only genes for structural bacterial microcompartment (BMC) proteins (Tph_c07040, Tph_c07020, Tph_c06980, Tph_c06960), an ethanolamine transporter (Tph_c07070) and an ethanolamine ammonia lyase (Tph_c06950, Tph_c06940) but also genes for enzymes that further degrade acetaldehyde. In *T. phaeum*, the cluster contains genes encoding proteins annotated as acetylating acetaldehyde dehydrogenase (Tph_c06970) and aldehyde:ferredoxin oxidoreductase (Tph_c07090). The *eut* cluster also contains an eutP gene (Tph_c06900), which was described as encoding an acetate kinase (Moore and Escalante-Semerena, 2016). This protein was not expressed and, moreover, the *eut* cluster does not contain genes for a phosphate acetyltransferase (eutD). A gene annotated as rnfC (Tph_c07030) and encoding a soluble protein containing iron–sulfur clusters was expressed as well, however no potential function and no relation of this single gene to a membrane-bound Rnf-complex that usually consists of six subunits was found. The gene cluster is putatively controlled by an AmiR and NasR Transcriptional Antiterminator Regulator domain (ANTAR) (Tph_c06910, Tph_c06920, and tph_c07090) as described for *E. faecalis* (Del Papa and Perego, 2008; Fox et al., 2009; Kaval and Garsin, 2018).

### Electron Microscopy

**FIGURE 2:**
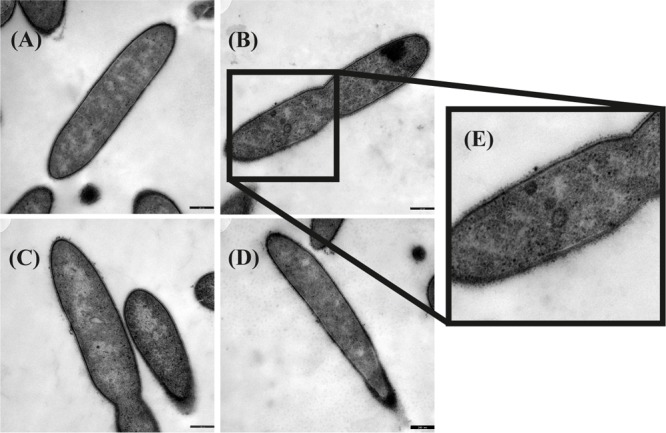
Transmission electron microscopic images of *Thermacetogenium phaeum* grown with different substrates. Fixation was performed with 2.5% glutardialdehyde, 2% OsO_4_ stain and 0.15% ruthenium red stain. **(A)** Syntrophic growth with ethanol. **(B)** Syntrophic growth with ethanolamine. **(C)** Axenic growth with methanol. **(D)** Syntrophic growth with acetate. **(E)** Twofold increased magnification showing irregularly-shaped electron dense structures that were interpreted as microcompartments. Bars represent 250 nm.

Transmission electron microscopy was performed to examine the presence of microcompartments during growth with different substrates. Ruthenium-red staining was performed in addition to OsO_4_ staining to visualize peptidoglycan structures like murein. Although initially thought to stain the cytoplasmic membrane (Cheng and Costerton, 1977; Fassel et al., 1992; Fassel and Edmiston, 1999), ruthenium-red helped to highlight also microcompartments (Supplementary Figure 7). In a culture grown with ethanolamine, irregularly-shaped electron-dense structures were observed (Figure 2). These structures were interpreted as microcompartment structures as similar structures were visualized by TEM before in *Salmonella enterica* after growth with ethanolamine (Crowley et al., 2008; Held et al., 2016). Such structures were not observed after growth with all other substrates, thus confirming the findings of the proteome analyses.

### Activities of Key Enzymes

Enzyme assays confirmed the results of the total proteome analysis. CO-dehydrogenase, hydrogenase, acetate kinase, and phosphate acetyltransferase displayed similar activities in cytoplasmic fractions obtained from cultures grown with all substrates (Table 2). The alcohol dehydrogenase displayed clear induction during growth with ethanolamine and ethanol. The aldehyde oxidoreductase was active during growth with all substrates but was induced 15-fold higher during growth with ethanol compared to methanol. Activity of the acetylating acetaldehyde dehydrogenase could be measured only in the cytoplasmic fraction of cells grown with ethanolamine. Formate dehydrogenase displayed activity during growth with all substrates but was induced fourfold higher after growth with ethanol and methanol than after growth with acetate. Activity of the methylene-THF reductase was assayed with benzyl viologen as oxidant in cytoplasmic and membrane fractions of cells grown with acetate. The activity of this enzyme was distributed equally in the membrane fraction (1.6 U/mg) and in the cytoplasmic fraction (1.6 U/mg). The activity is also present in methanol-grown cells. As the cultures grow fastest with methanol, this cultivation condition was used to grow cells for the investigation of the biochemical properties of methylene-THF reductase and methylene-THF dehydrogenase.

**Table 2:** Enzyme activities measured in the cytoplasmic fraction of *T. phaeum* grown with different substrates.

		Ethanol^a^	Ethanolamine^a^	Methanol^a^	Acetate^b^
Acetylating aldehyde DH		bd^c^	0.005 ± 0	bd	bd
Aldehyde oxidoreductase (AOR)	NAD^+^	0.027 ± 0.007	0.012 ± 0.001	0.005 ± 0.001	0.001 ± 0
	BV	35.4 ± 6.2	4.8 ± 0.800	2.3 ± 0.050	1.6 ± 0.342
	MV	10.3 ± 2.5	1.9 ± 0.205	0.751 ± 0.157	0.518 ± 0.298
Alcohol DH	NAD^+^	0.157 ± 0.004	0.337 ± 0.003	0.004 ± 0	0.004 ± 0.001
Phosphate acetyltransferase		27.6 ± 0.508	25.1 ± 1.9	50.4 ± 3.3	19.6 ± 1.3
Acetate kinase^d^		54.4	111.9	130.4	41.6
Formate DH	NAD^+^	bd	0.005 ± 0.001	0.058 ± 0.010	0.017 ± 0.001
	BV	39.8 ± 8.1	14.7 ± 1.0	39.7 ± 5.2	9.7 ± 1.1
	MV	9.3 ± 1.6	3.9 ± 0.4	10.0 ± 1.3	3.4 ± 0.400
Hydrogenase	BV	0.750 ± 0.135	0.815 ± 0.2	0.434 ± 0.175	1.5 ± 0.313
	MV	0.503 ± 0.052	0.179 ± 0.022	0.175 ± 1.3	0.465 ± 0.055
CO DH	BV	0.723 ± 0.203	2.7 ± 0.253	1.3 ± 0.198	1.7 ± 0.175
	MV	0.584 ± 0.158	2.4 ± 0.255	1.1 ± 0.186	1.5 ± 0.179

*All activities were measured at least in triplicates and are given in Units per mg protein. All enzyme assays were performed in triplicates. ^*a*^Axenic culture. ^*b*^Syntrophic culture. ^*c*^bd: below detection limit (>1 mU per mg protein). ^*d*^For acetate kinase no standard deviation was calculated as it was a discontinuous measurement.*

Activity of methylene-THF reductase was assayed in the direction of methyl-THF formation with NADH as reductant or in the direction of methylene-THF formation with methyl-THF as substrate and BV as artificial electron acceptor in cytoplasmic and membrane fractions of *T. phaeum* cells grown with methanol and *A. woodii* cells grown with fructose. The activity with NADH as electron donor was detectable in the cytoplasmic fraction of *T. phaeum* only after a purification step with Q-Sepharose (Table 3). An activity of 21 ± 11 mU was found in fraction 2 that eluted with 1 M NaCl. In the cytoplasmic fraction of *A. woodii* an activity of 1519 ± 70 could be directly measured but it increased after purification (1 M NaCl, fraction 2). The membrane fractions of both bacteria showed similar activities of 39 ± 15 mU (*T. phaeum*) and 43 ± 15 mU (*A. woodii*). With benzyl viologen as electron acceptor, no activity could be measured in cytoplasmic fractions of *A. woodii*. In *T. phaeum*, however, an activity of the methylene-THF reductase was measured in the cytoplasmic fraction that was enriched in fraction 1. Interestingly, the activity in the membrane fraction of *T. phaeum* was higher compared to the cytoplasmic fraction, while the activity was much lower in *A. woodii*.

**Table 3:** Methylene-THF reductase activity in *T. phaeum* and *A. woodii*.

	Substrate	Electron carrier	Activity [mU]			
			CF	Fraction 1	Fraction 2	MF
***T. phaeum***						
	**Methyl-THF**	**BV**	148 ± 49	bd^a^	245 ± 63	826 ± 177
	**Methylene-THF**	**NADH**	bd^a^	bd^a^	21 ± 11	39 ± 15
	**Formaldehyde control**	**NADH**	1 ± 0	bd^a^	bd^a^	bd^a^
***A. woodii***						
	**Methyl-THF**	**BV**	bd^a^	bd^a^	2226 ± 343	9 ± 2
	**Methylene-THF**	**NADH**	1519 ± 70	462 ± 43	8371 ± 1272	43 ± 22
	**Formaldehyde control**	**NADH**	bd^a^	bd^a^	bd^a^	bd^a^

*Enzyme activities were measured in the cytoplasmic fraction (CF) and membrane fraction (MF) of the two organisms. The cytoplasmic fraction was enriched by Q sepharose chromatography. The fraction that eluted with 200 mM NaCl was named fraction 1, and the fraction that eluted with 1 M NaCl was named fraction 2. **^*a*^**Below detection limit (<1 mU per mg protein).*

Methylene-THF dehydrogenase was assayed with NAD^+^ as electron acceptor in purified fractions of the cytoplasmic fraction. Activity was high in the fraction 1 that eluted with 200 mM NaCl from the column (Table 4). Here, a similar fractionation pattern was observed for *A. woodii*.

**Table 4:** Methylene-THF dehydrogenase activity in *T. phaeum* and *A. woodii*.

	Substrate	Electron carrier	CF	Fraction 1	Fraction 2	MF
***T. phaeum***						
	**Methylene-THF**	**NAD^+^**	^a^	240792 ± 15292	735 ± 299	^a^
	**Formaldehyde control**	**NAD^+^**	7 ± 1	bd^b^	11	bd^b^
***A. woodii***						
	**Methylene-THF**	**NAD^+^**	^a^	67756 ± 4221	275 ± 96	^a^
	**Formaldehyde control**	**NAD^+^**	bd^b^	bd^b^	bd^b^	bd^b^

*Enzyme activities were measured in the cytoplasmic fraction (CF) and membrane fraction (MF) of the two organisms. The cytoplasmic fraction was enriched by Q sepharose chromatography. The fraction that eluted with 200 mM NaCl was named fraction 1, and the fraction that eluted with 1 M NaCl was named fraction 2. ^*a*^Not measured. ^*b*^Below detection limit (<1 mU per mg protein).*

## Discussion

### Ethanol and Ethanolamine Degradation

This study analyzed growth and substrate transformation by *T. phaeum* with ethanol and ethanolamine to investigate the route of degradation through the intermediate acetaldehyde. There are two options for anaerobic acetaldehyde oxidation: in the pathway via the acetylating acetaldehyde dehydrogenase, phosphate acetyltransferase, and acetate kinase, one ATP is gained and NAD^+^ is reduced. Alternatively, acetaldehyde is oxidized to acetate via acetaldehyde:ferredoxin oxidoreductase, and energy must be conserved via alternative mechanisms, e.g., by electron transport phosphorylation. The acetogen *A. woodii* degrades ethanol to acetate via the acetate kinase pathway and disposes of the gained electrons via the Wood–Ljungdahl pathway, reducing CO_2_ to acetate. The ethanol-to-acetate ratio of this reaction is 2:3, and 0.55 ATP per mole ethanol is generated in *A. woodii* (Bertsch et al., 2016):

(1) $$\begin{array}{c} 2{\mathrm{CH}}_{3}{\mathrm{CH}}_{2}\mathrm{OH}+2{\mathrm{CO}}_{2}\to 3{\mathrm{CH}}_{3}{\mathrm{COO}}^{-}+3H^{+}\Delta G^{{}^{\circ}}{}^{\prime }\;=\;-76 kJ per reaction \end{array}$$

**FIGURE 3:**
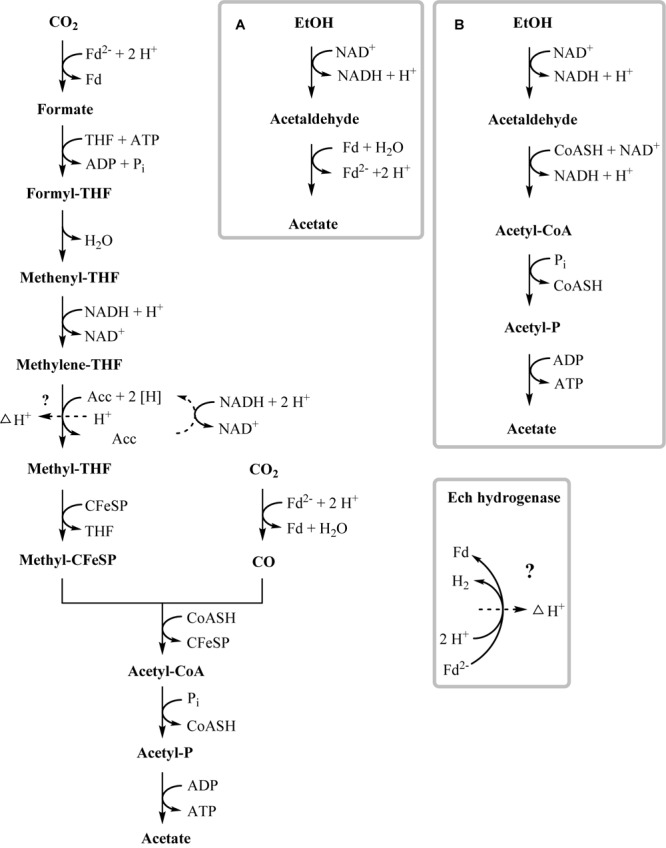
Proposed pathway of ethanol degradation in *T. phaeum*. Electrons originating from ethanol oxidation are disposed via the Wood–Ljungdahl pathway by CO_2_ reduction to acetate. ATP generation most likely occurs by formation of a proton gradient across the membrane (indicated by dashed arrow). Gray frames depict possible functional units of enzymes. **(A)** High affinity ethanol degradation and **(B)** low affinity ethanol degradation. However, production of minor amounts of ATP via acetaldehyde and acetate kinase cannot be ruled out.

In the present work, *T. phaeum* grew in axenic culture with ethanol with nearly the same overall reaction stoichiometry of 27 mM ethanol converted to 35 mM acetate, which includes additional acetate production from CO_2_ through the Wood–Ljungdahl pathway. In syntrophic culture, the stoichiometry was 39 mM ethanol to 45 mM acetate, and this nearly 1:1 ethanol-to-acetate ratio reflects release of excess reducing equivalents as methane by the syntrophic partner (Table 1). Axenic growth of *T. phaeum* with 40 mM ethanol was poor (OD = 0.12) compared to *A. woodii*, which reached an OD of more than 0.8 with 50 mM ethanol (Bertsch et al., 2016). A key reaction in the Wood–Ljungdahl pathway is the reduction of CO_2_ to bound CO in the CO dehydrogenase reaction (E°’ = -520 mV), which requires reduced ferredoxin as a reductant. *A. woodii* uses an Rnf complex to lift electrons from the electron potential of NADH oxidation to the electron potential of ferredoxin (Bertsch et al., 2016). This option is not available in *T. phaeum* as there is no Rnf complex encoded in the genome (Oehler et al., 2012). The poor growth yield (1.2 g per mol substrate) indicates that acetaldehyde derived from ethanol oxidation is oxidized directly to acetate via acetaldehyde:ferredoxin oxidoreductase. This hypothesis is substantiated by the 1000-fold increase in aldehyde:ferredoxin oxidoreductase (Tph_c08220) abundance compared to the acetylating acetaldehyde dehydrogenase (Tph_c06970) during growth on ethanol (Figure 1). No activity was detected for the acetylating acetaldehyde dehydrogenase (Table 2) although it was found to be expressed in the proteome (Figure 1). Lack of measurable activity in the cytoplasmic fraction could be due to the presence of alcohol dehydrogenase, i.e., reduction of acetaldehyde to ethanol which would consume the NADH formed in acetaldehyde oxidation. However, when comparing abundance and activity of acetaldehyde:ferredoxin oxidoreductase to the abundance and activity of acetylating acetaldehyde dehydrogenase, the latter seems to be negligibly low and cannot contribute much to the overall substrate turnover. Therefore, only little ATP can be supplied through the acetate kinase reaction in axenic culture. In syntrophic co-culture, abundance of acetylating acetaldehyde dehydrogenase (Tph_c06970) is much higher than in axenic culture. The NADH electrons might be disposed of through NADH-dependent formate dehydrogenase or hydrogenase for interspecies electron transfer. As a consequence, more ATP can be gained by substrate-level phosphorylation as no reversed electron transport or ferredoxin-coupled acetaldehyde oxidation is necessary. This is also reflected by the detected higher growth yield of 1.8 g per mol substrate in syntrophic co-culture versus 1.2 g per mol substrate in axenic culture. However, it is still unclear how energy is conserved during axenic growth on ethanol, as neither ethanol oxidation itself nor the Wood–Ljungdahl pathway yield net ATP (Figure 3).

**FIGURE 4:**
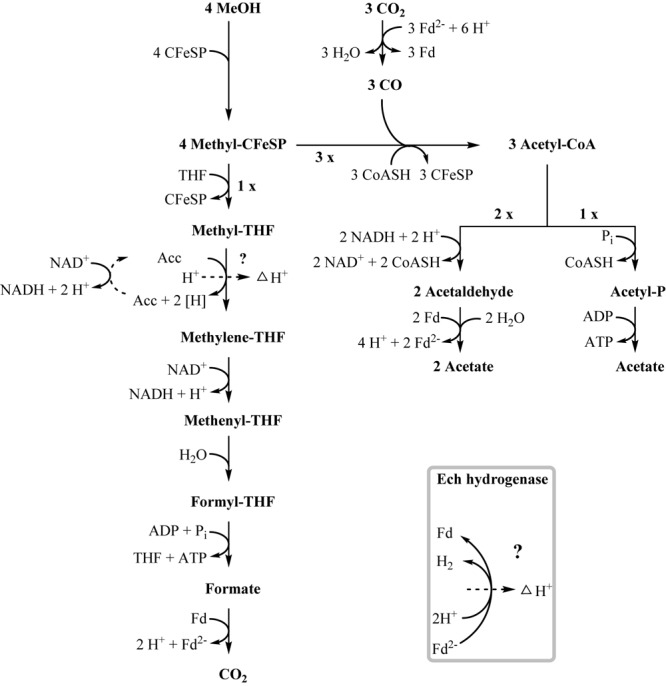
Proposed pathway of methanol degradation in *T. phaeum*. The pathway resembles the pathway proposed for *Acetobacterium woodii* but with modifications as mentioned in the text. One methanol is oxidized to CO_2_ to generate electrons. The enzyme system coupling NAD^+^-reduction to methyl-THF oxidation and proton translocation is unknown. Aldehyde:ferredoxin oxidoreductase is used to regenerate reduced ferredoxin.

One option to conserve energy during acetogenesis is the methylene-THF reductase (Tph_c15100–Tph_c15110) reaction with a redox potential of E° = -200 mV (Bertsch et al., 2015). Ethanol oxidation has a similar redox potential (-190 mV), and when coupled to NAD^+^ reduction (-320 mV) the equilibrium is on the side of ethanol. Through maintaining a permanently low acetaldehyde concentration by a highly affine acetaldehyde:ferredoxin oxidoreductase, the equilibrium of ethanol oxidation could be shifted toward acetaldehyde and NADH formation. With this, re-oxidation of NADH with methylene-THF would become exergonic and could provide the necessary reaction enthalpy for energy conservation. If this reaction could be coupled to proton gradient formation, e.g., through a quinone cycle, energy could be conserved by ATP synthase. This system would yield a fraction of an ATP equivalent per ethanol oxidized, in accordance with the low growth yield of 1.2 g per mol substrate. However, an enzyme system responsible for quinone-linked proton translocation in the cytoplasmic membrane has not been identified yet. Interaction of methylene-THF reduction with the membrane is indicated by enzyme activity assays with membrane fractions of syntrophic co-cultures grown with acetate, where a membrane-associated enzyme system working in reverse was detected. This membrane-bound activity was identified also in methanol-grown cells. The activity of methyl-THF oxidation with benzyl viologen was located both in the membrane and in the cytoplasmic fraction at similar quantities, indicating that this enzyme is only loosely attached to the membrane. The physiological electron acceptor of this reaction is still unknown, yet these results indicate that at least one membrane-bound enzyme is involved in the Wood–Ljungdahl-pathway of *T. phaeum* (Figure 3, 4). The linkage of the methylene-THF reductase to a quinone cycle could be heterodisulfide reductase A (Tph_c15090), which is encoded in the gene cluster of the Wood–Ljungdahl pathway. Subunits B and C are encoded in a gene cluster together with a membrane-bound formate dehydrogenase (Tph_c15370-15410) and with genes for quinone synthesis (Tph_c15430-15450), and presence of menaquinone-7 has been reported already in the original description of this bacterium (Hattori et al., 2000).

Growth with ethanolamine has not been studied yet in acetogens; however, *A. woodii* is able to grow with ethylene glycol, which is degraded in a similar way as ethanolamine (Trifunović et al., 2016). Ethylene glycol is first dehydrated to acetaldehyde, which is then dismutated to ethanol and acetate, with subsequent ethanol oxidation. During growth with ethylene glycol, *A. woodii* exhibits diauxic growth with intermediate accumulation of ethanol. Growth of *T. phaeum* with ethanolamine was not diauxic, neither in axenic nor in syntrophic co-culture. In axenic cultures, ethanol accumulated to 6 mM concentration and was not depleted further. The growth yield of axenic cultures with ethanolamine was 1.6 g per mole substrate whereas syntrophic cultures growing with ethanolamine yielded 3.0 g per mole substrate. This again demonstrates the difficulties of the axenic culture to dispose of electrons from ethanol oxidation.

Three different types of ethanolamine utilization (*eut*) gene clusters coding for microcompartments (BMCs) have been described so far (Tsoy et al., 2009; Kaval and Garsin, 2018). *T. phaeum* carries a comparably extensive *eut* cluster which is controlled by an Antiterminator regulatory (ANTAR) system as described for *E. faecalis* (Del Papa and Perego, 2008; Fox et al., 2009; Ramesh et al., 2012). Here, expression is post-transcriptionally regulated by the substrate ethanolamine and AdoCobl, the cofactor of ethanolamine ammonia lyase (Fox et al., 2009). This means that expression of the BMC genes is induced by the presence of ethanolamine rather than by acetaldehyde. The proteome data revealed specific induction of proteins of the eut cluster during growth with ethanolamine, compared to the other substrates. This confirms the presumption that the regulation is comparable to that in *E. faecalis*. These genes were expressed at a basal level during growth with all other substrates, which is most likely due to leaky expression. The presence of microcompartments was confirmed by TEM images, which exhibited irregularly-shaped structures in cells grown with ethanolamine but not in cells grown with any other substrate (Figure 2). The toxic intermediate acetaldehyde plays a role in the degradation of ethanolamine, but also of ethanol and methanol. The pool size of acetaldehyde during ethanol and methanol degradation is expected to be very low as a consequence of the reaction equilibria of ethanol dehydrogenase, acetaldehyde:ferredoxin oxidoreductase, and acetylating aldehyde dehydrogenase. Only the ethanolamine ammonia lyase reaction is far on the side of acetaldehyde production and might lead to its accumulation to toxic levels. Consequently, microcompartments are produced only during growth with ethanolamine to avoid toxic acetaldehyde concentrations in the cytoplasm.

### Methanol Degradation

In methanol degradation by *A. woodii* (Kremp et al., 2018), the mta operon comprises the genes responsible for the demethylation of methanol. This gene cluster contains eight genes: sensory proteins, transcriptional regulators, a corrinoid protein, and methyltransferase I and II. In *T. phaeum* this gene cluster encodes only four proteins. A methanol:corrinoid methyltransferase was induced during growth with methanol (Tph_c03590). Two methyltetrahydrofolate-homocysteine methyltransferases (Tph_c03600–Tph_c03610) are located in the same gene cluster and are also induced during growth with methanol. One of these proteins (Tph_c03600) has presumably been annotated wrongly and is actually a corrinoid protein. In *A. woodii*, 60 mM methanol supported growth to a final OD of 1.5 with a doubling time of 16 h. At an initial concentration of 40 mM methanol, growth of *T. phaeum* was slightly faster with a doubling time of 12.5 h. However, the final OD amounted only to 0.64, which is substantially less than expected. As in *A. woodii*, the stoichiometry of methanol conversion to acetate is 4:3 (Table 1), but the ATP yield appears to be different (Figure 4). Synthesis of three acetyl residues through the Wood–Ljungdahl pathway requires reduction of three CO_2_ to bound CO in the CODH/ACS reaction (E°’ = -520 mV). Oxidation of one methyl group via the reversed Wood–Ljungdahl pathway delivers 2 NADH and one reduced ferredoxin. Enzyme tests in protein preparations of *T. phaeum* described above demonstrate that NADH does not serve as an efficient electron carrier for methylene-THF reductase when assayed in the direction of methyl-THF formation. Instead, high activities with benzyl viologen as electron acceptor indicate that a different, yet unidentified electron acceptor must be responsible for the methylene-THF reductase activity. This finding is not surprising, as the NADH-binding, neighboring subunit of the metV/metF genes is missing in *T. phaeum* as opposed to the respective operon structure in *A. woodii* (Bertsch et al., 2015). It remains pure speculation whether the wrongly annotated rnfC gene (Tph_c07030) located elsewhere in the genome is a possible homolog of the third methylene-THF reductase subunit in *A. woodii*, wrongly annotated as electron transport complex protein RnfC2. The latter is the NADH-binding subunit of the methylene-THF reductase in *A. woodii* but is not related to the Rnf-complex. *A. woodii* uses its Rnf complex to generate reduced ferredoxin from NADH at the expense of a sodium ion gradient supplied by ATP hydrolysis. As no Rnf complex is encoded in the genome of *T. phaeum*, reduced ferredoxin must be gained by other means. Fermentation of methyl groups via acetyl-CoA to acetaldehyde and final oxidation to acetate could release reduced ferredoxin (Figure 4). In the present study, *in vitro* activity of acetaldehyde:BV oxidoreductase was demonstrated in methanol-grown cells. Assuming that benzyl viologen mimics the electron acceptor function of ferredoxin, this enzyme could generate reduced ferredoxin to feed the CODH reaction (Figure 4). The two NADH necessary for the acetylating acetaldehyde dehydrogenase, one ferredoxin and one ATP, can be obtained by oxidation of one methyl group to CO_2_. Here, only one methanol is converted to acetate via the acetate kinase pathway and thus yields one ATP. Overall, 0.5 ATP could be gained per conversion of one methanol to acetate (Figure 4). This corresponds well to the growth yield calculated in this study, which amounts to 4.5 g dry mass per mole methanol, assuming an energy yield coefficient of 10 g cell dry mass per mole of ATP (Payne, 1970). For *A. woodii*, 0.625 ATP per mole methanol was calculated (Kremp et al., 2018). The calculated Gibb’s free energy of the conversion of methanol to acetate amounts to -212 kJ per reaction run (-53 kJ per mole methanol) and thus corresponds to 0.66 mole of ATP per mole methanol (Eq. 2) (Schink, 1997).

(2) $$\begin{array}{c} 4{\mathrm{CH}}_{3}\mathrm{OH}+2{\mathrm{CO}}_{2}\to 3{\mathrm{CH}}_{3}{\mathrm{COO}}^{-}+3H^{+}+2H_{2}O\Delta G^{{}^{\circ}}{}^{\prime }=-212 kJ per reaction \end{array}$$

### Acetate Oxidation

The pathway of acetate oxidation by thermophilic fermenting bacteria has not yet been completely elucidated. For *Syntrophaceticus schinkii*, the species most closely related to *T. phaeum* known so far, a new pathway in addition to the Wood–Ljungdahl pathway was suggested on the basis of genomic data. This pathway involves activation of acetate to acetyl-CoA and addition of formate and ammonium to form serine (Nobu et al., 2015; Manzoor et al., 2016). After splitting off ammonia, a glycine cleavage system transfers a methenyl group to THF. All enzymes necessary for this pathway are encoded in the genome of *T. phaeum*, but none of them was induced during growth with acetate. Thus, the proposed pathway is not used by *T. phaeum* during syntrophic growth with acetate.

In recent publications it was proposed that acetate can be activated via aldehyde:ferredoxin oxidoreductase to acetaldehyde and further to ethanol (Köpke et al., 2010; Bengelsdorf et al., 2013). No ATP investment is needed in this pathway. Our proteome data indicate that in cells grown with acetate, an aldehyde:ferredoxin oxidoreductase (Tph_c08220) is expressed. This finding was substantiated by *in vitro* enzyme assays, which displayed an activity of 1.6 U per mg protein with benzyl viologen as artificial electron acceptor. This suggests an alternative path of acetate activation without ATP investment in this step as proposed for *C. ljungdahli* and other microorganisms (Köpke et al., 2010; Bengelsdorf et al., 2013). The “saved” ATP could facilitate energy investment in the unfavorable oxidation of methyl-THF with NAD^+^. Alternatively, or additionally, the subsequent highly affine and exergonic oxidation of methylene-THF with NAD^+^ could shift the thermodynamic equilibrium such that methyl-THF oxidation is enabled (Bertsch et al., 2015; Kremp et al., 2018). Yet, acetate kinase (Tph_c10090) and phosphate acetyltransferase (Tph_c10080) were expressed as well and were detectable in enzyme assays. The presence of these enzymes could indicate an activation of acetate by phosphorylation rather than direct reduction, but this reaction path might also be used to generate acetyl-CoA for anabolic processes. This alternative mode of acetate activation is subject to further investigations.

## Conclusion

The absence of an Rnf complex in *T. phaeum* necessitates alternative pathways for acetogenic degradation of methanol and ethanol, different from those used by *A. woodii*. Evidence from proteome analysis showed that net energy conservation during axenic growth on ethanol cannot be accomplished by substrate-level phosphorylation alone and is most likely achieved by electron transport phosphorylation. Proteome data and TEM showed that in *T. phaeum*, microcompartments are expressed only during growth with ethanolamine. This finding proves that microcompartments are not obligatory for growth with ethanol and for protecting the cell from the toxic intermediate acetaldehyde, although the latter occurs during growth with all three substrates. Hence the acetaldehyde concentration needs to be kept low by subsequent oxidation reactions during ethanol, methanol, and putatively acetate degradation. Proteome data and enzyme assays propose an alternative pathway of acetate activation during acetate oxidation. Using this hypothetical activation, the ATP investment by acetate kinase in the first step could be circumvented. This approach would solve the problem of energy conservation in *T. phaeum* and is therefore the subject of current biochemical investigations.

## Data Availability

All data referred to is included in the article.

## Author Contributions

AK conducted experiments designed by AK and NM. AK, NM, and BS wrote and approved the final manuscript.

## Conflict of Interest Statement

The authors declare that the research was conducted in the absence of any commercial or financial relationships that could be construed as a potential conflict of interest.
