# Supplementary material for: Alternative Pathways of Acetogenic Ethanol and Methanol Degradation in the Thermophilic Anaerobe Thermacetogenium phaeum
Source: Front Microbiol. 2019 Mar 19;10:423. doi: 10.3389/fmicb.2019.00423 (PMC6436200; doi:10.3389/fmicb.2019.00423)
Supplement: Supplementary file 1 [file Image_1.pdf]

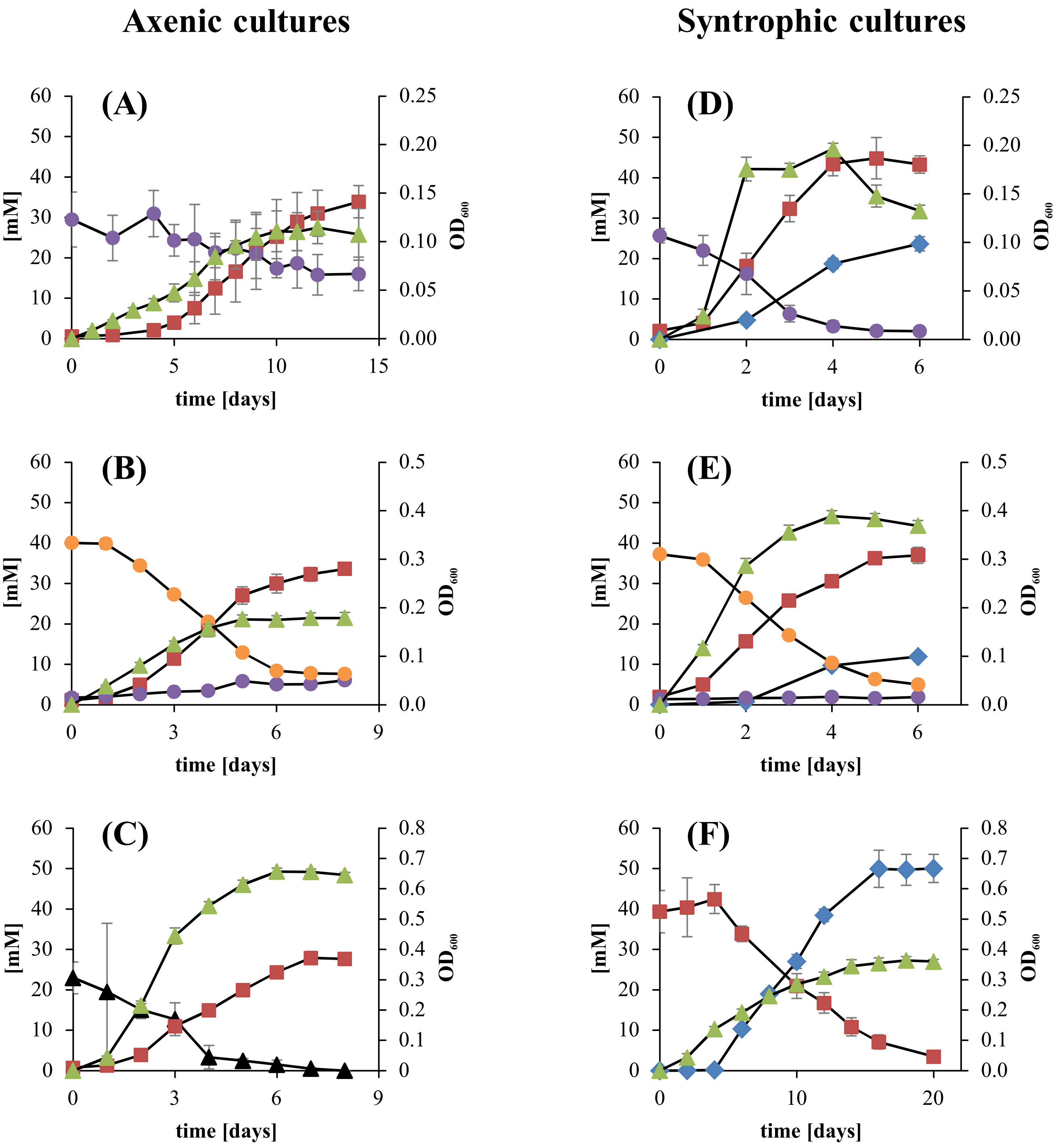

Supplementary Figure 1: Growth curves of *Thermacetogenium phaeum* depicting substrate depletion, product formation and OD_{600} increase. **(A)** axenic growth with ethanol. **(B)** axenic growth with ethanolamine. **(C)** axenic growth with methanol. **(D)** syntrophic growth with ethanol. **(E)** syntrophic growth with ethanolamine. **(F)** syntrophic growth with acetate. Green triangles depict OD_{600}, red squares depict acetate concentration, purple circles depict ethanol concentration, black triangles depict methanol concentration, orange circles depict ethanolamine concentation, blue diamonds depict methane concentration. All concentrations are given in mM +/- standard deviation; some error bars are smaller than symbol size.
