# Supplementary material for: Alternative Pathways of Acetogenic Ethanol and Methanol Degradation in the Thermophilic Anaerobe Thermacetogenium phaeum
Source: Front Microbiol. 2019 Mar 19;10:423. doi: 10.3389/fmicb.2019.00423 (PMC6436200; doi:10.3389/fmicb.2019.00423)
Supplement: Supplementary file 3 [file Image_3.pdf]

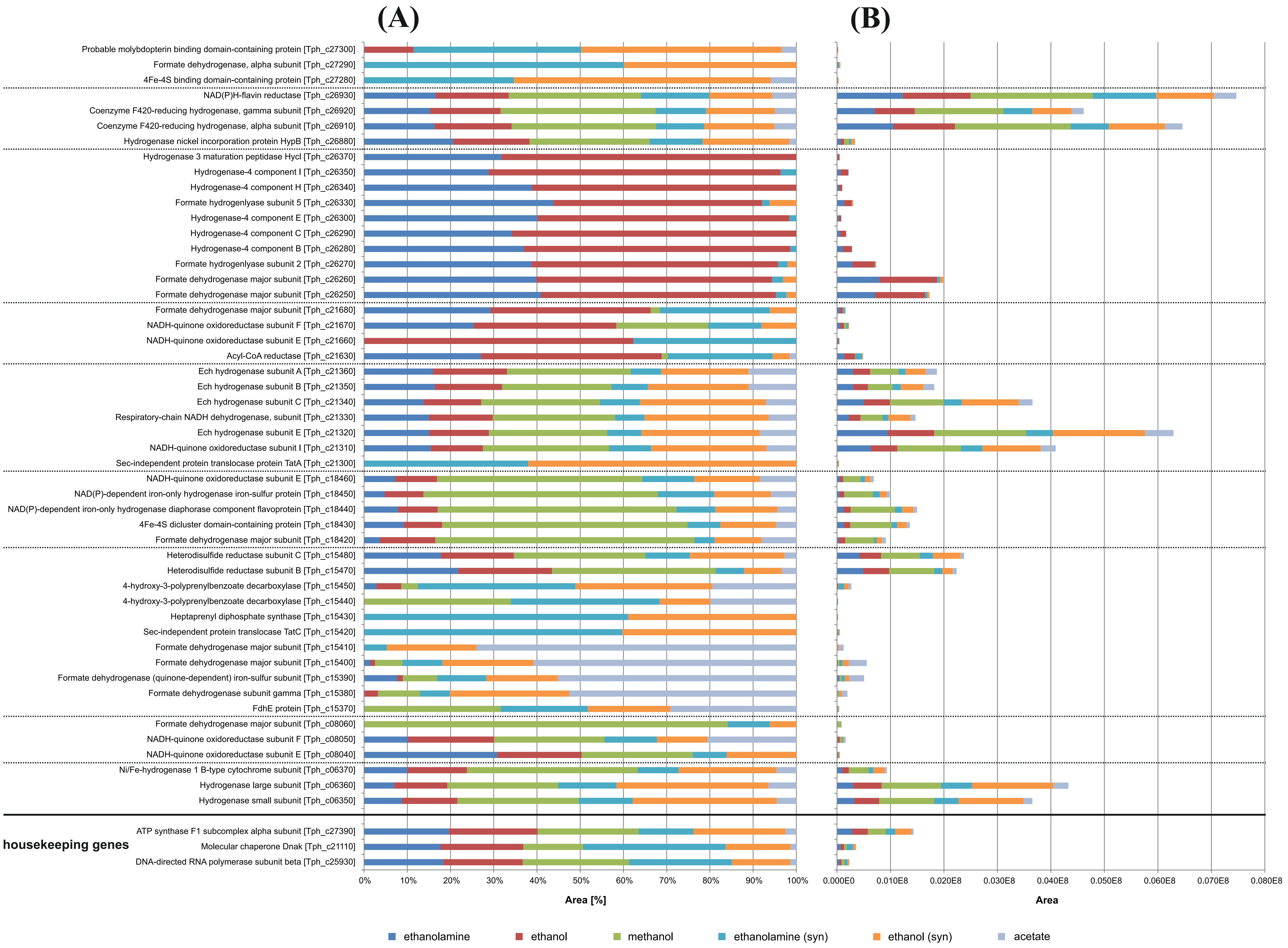
(A)
(B)
Probable molybdopterin binding domain-containing protein [Tph_c27300]
Formate dehydrogenase, alpha subunit [Tph_c27290]
4Fe-4S binding domain-containing protein [Tph_c27280]
NAD(P)H-flavin reductase [Tph_c26930]
Coenzyme F420-reducing hydrogenase, gamma subunit [Tph_c26920]
Coenzyme F420-reducing hydrogenase, alpha subunit [Tph_c26910]
Hydrogenase nickel incorporation protein HypB [Tph_c26880]
Hydrogenase 3 maturation peptidase HycI [Tph_c26370]
Hydrogenase-4 component I [Tph_c26350]
Hydrogenase-4 component H [Tph_c26340]
Formate hydrogenlyase subunit 5 [Tph_c26330]
Hydrogenase-4 component E [Tph_c26300]
Hydrogenase-4 component C [Tph_c26290]
Hydrogenase-4 component B [Tph_c26280]
Formate hydrogenlyase subunit 2 [Tph_c26270]
Formate dehydrogenase major subunit [Tph_c26260]
Formate dehydrogenase major subunit [Tph_c26250]
Formate dehydrogenase major subunit [Tph_c21680]
NADH-quinone oxidoreductase subunit F [Tph_c21670]
NADH-quinone oxidoreductase subunit E [Tph_c21660]
Acyl-CoA reductase [Tph_c21630]
Ech hydrogenase subunit A [Tph_c21360]
Ech hydrogenase subunit B [Tph_c21350]
Ech hydrogenase subunit C [Tph_c21340]
Respiratory-chain NADH dehydrogenase, subunit [Tph_c21330]
Ech hydrogenase subunit E [Tph_c21320]
NADH-quinone oxidoreductase subunit I [Tph_c21310]
Sec-independent protein translocase protein TatA [Tph_c21300]
NADH-quinone oxidoreductase subunit E [Tph_c18460]
NAD(P)-dependent iron-only hydrogenase iron-sulfur protein [Tph_c18450]
NAD(P)-dependent iron-only hydrogenase diaphorase component flavoprotein [Tph_c18440]
4Fe-4S dicluster domain-containing protein [Tph_c18430]
Formate dehydrogenase major subunit [Tph_c18420]
Heterodisulfide reductase subunit C [Tph_c15480]
Heterodisulfide reductase subunit B [Tph_c15470]
4-hydroxy-3-polyprenylbenzoate decarboxylase [Tph_c15450]
4-hydroxy-3-polyprenylbenzoate decarboxylase [Tph_c15440]
Heptaprenyl diphosphate synthase [Tph_c15430]
Sec-independent protein translocase TatC [Tph_c15420]
Formate dehydrogenase major subunit [Tph_c15410]
Formate dehydrogenase major subunit [Tph_c15400]
Formate dehydrogenase (quinone-dependent) iron-sulfur subunit [Tph_c15390]
Formate dehydrogenase subunit gamma [Tph_c15380]
FdhE protein [Tph_c15370]
Formate dehydrogenase major subunit [Tph_c08060]
NADH-quinone oxidoreductase subunit F [Tph_c08050]
NADH-quinone oxidoreductase subunit E [Tph_c08040]
Ni/Fe-hydrogenase 1 B-type cytochrome subunit [Tph_c06370]
Hydrogenase large subunit [Tph_c06360]
Hydrogenase small subunit [Tph_c06350]
housekeeping genes
ATP synthase F1 subcomplex alpha subunit [Tph_c27390]
Molecular chaperone Dnak [Tph_c21110]
DNA-directed RNA polymerase subunit beta [Tph_c25930]
0% 10% 20% 30% 40% 50% 60% 70% 80% 90% 100%
Area [%]
0.000E0 0.010E8 0.020E8 0.030E8 0.040E8 0.050E8 0.060E8 0.070E8 0.080E8
Area
ethanolamine
ethanol
methanol
ethanolamine (syn)
ethanol (syn)
acetate

Supplementary Figure 3: Proteome data of all expressed hydrogenases and formate dehydrogenases obtained during growth of *Thermacetogenium phaeum* with different substrates. All expressed genes of the different gene clusters are shown in the graph. The clusters were separated by dashed lines. Housekeeping genes were depicted beneath the solid line. **(A)**: normalized data: percentage of protein abundances (expressed as area of mass spectroscopy signals) under various growth conditions relative to the summed protein abundances (area) of all growth conditions. **(B)**: absolute sums of area values of the respective identified proteins obtained in all growth conditions are shown here.
