# Supplementary material for: Alternative Pathways of Acetogenic Ethanol and Methanol Degradation in the Thermophilic Anaerobe Thermacetogenium phaeum
Source: Front Microbiol. 2019 Mar 19;10:423. doi: 10.3389/fmicb.2019.00423 (PMC6436200; doi:10.3389/fmicb.2019.00423)
Supplement: Supplementary file 5 [file Image_5.pdf]

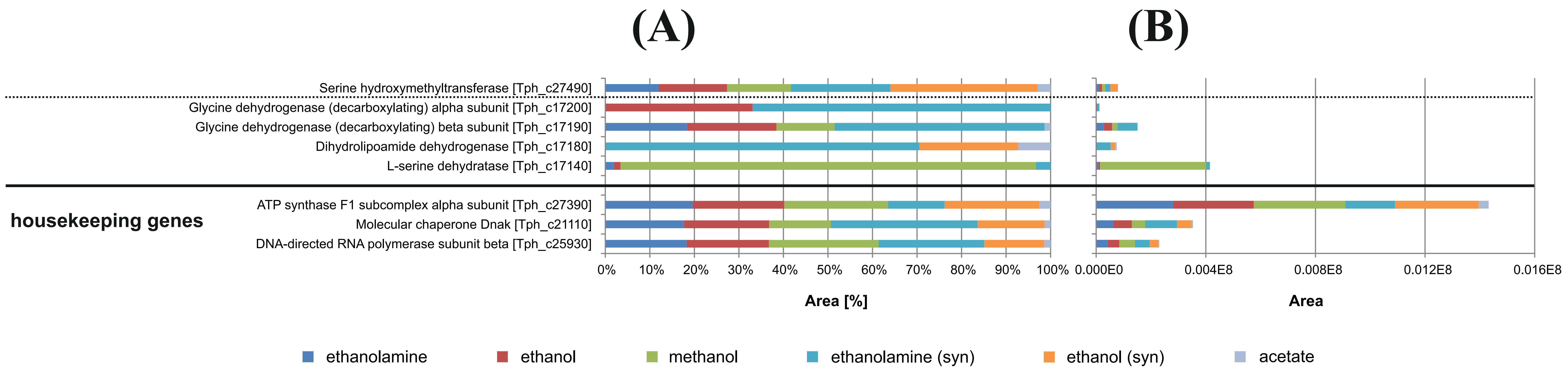

Supplementary Figure 5: Proteome data of the serine hydroxymethyltransferase and the glycine cleavage system obtained during growth of *Thermacetogenium phaeum* with different substrates. Proteins located in different clusters were separated by dashed lines. Housekeeping genes were depicted beneath the solid line. **(A)**: normalized data: percentage of protein abundances (expressed as area of mass spectroscopy signals) under various growth conditions relative to the summed protein abundances (area) of all growth conditions. **(B)**: absolute sums of area values of the respective identified proteins obtained in all growth conditions are shown here.
