# Supplementary material for: Alternative Pathways of Acetogenic Ethanol and Methanol Degradation in the Thermophilic Anaerobe Thermacetogenium phaeum
Source: Front Microbiol. 2019 Mar 19;10:423. doi: 10.3389/fmicb.2019.00423 (PMC6436200; doi:10.3389/fmicb.2019.00423)
Supplement: Supplementary file 7 [file Image_7.pdf]

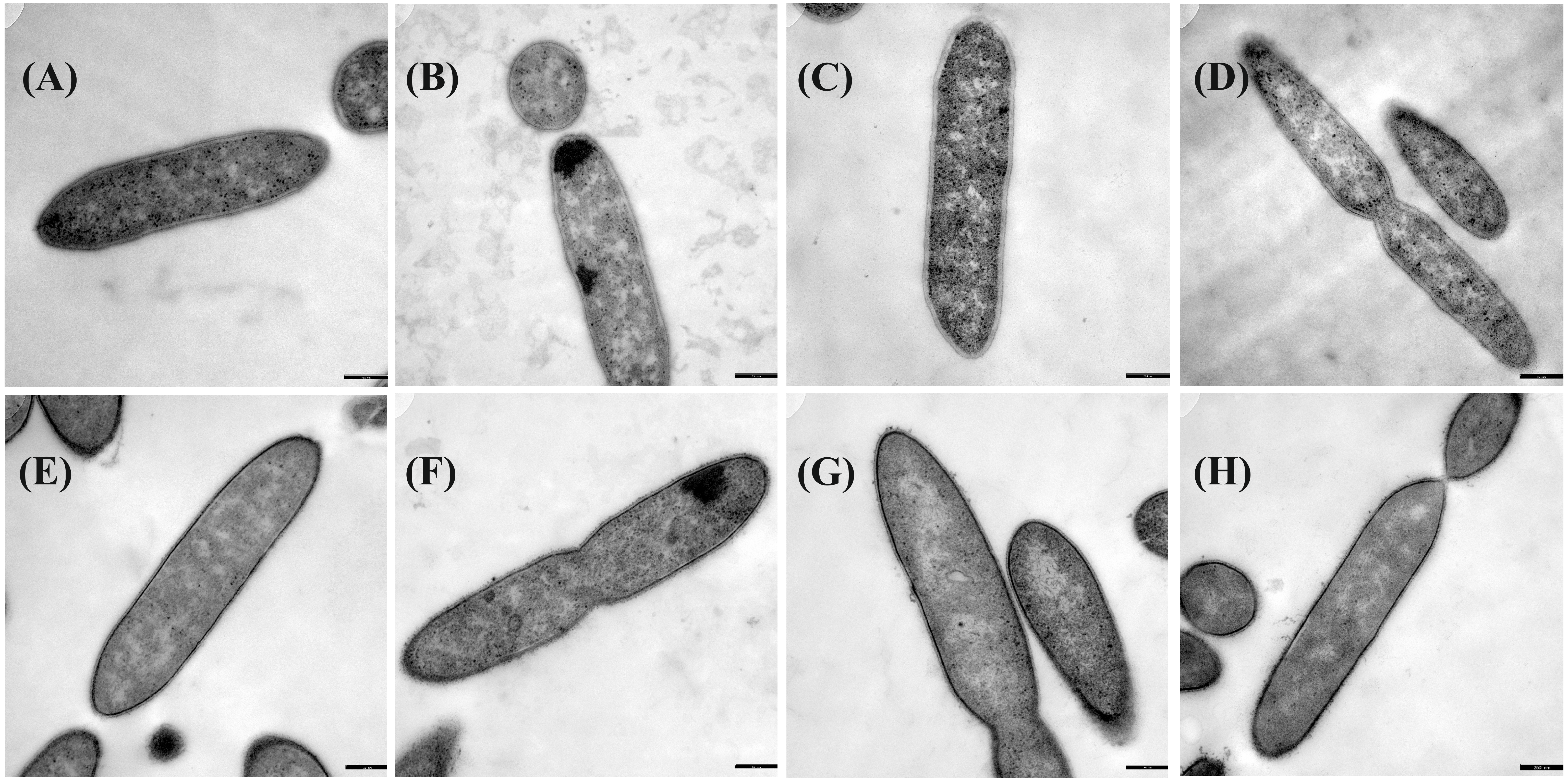

Supplementary Figure 7: Transmission electron microscopy pictures of *Thermacetogenium phaeum* grown with different substrates. **(A)**/**(E)** syntrophic growth with ethanol. **(B)**/**(F)** syntrophic growth with ethanolamine. Irregular shaped electron dense circles in **(F)** were interpreted to be microcompartments. **(C)**/**(G)** axenic growth with methanol. **(D)**/**(H)** syntrophic growth with acetate. **(A)**-**(D)** Fixation with 2.5% glutaraldehyde, 2% OsO4 stain. **(E)**-**(H)** Additional 0.15% ruthenium red stain. Bars indicate 250 nm.
